# Awareness, attitudes, and barriers toward Transthyretin Amyloid Cardiomyopathy in Latin America: A questionnaire-based cross-sectional study

**DOI:** 10.1371/journal.pone.0351224

**Published:** 2026-06-26

**Authors:** Cecilia Camacho-Hubner, Marcia Waddington-Cruz, María Juliana Rodríguez-González, Marcus Vinicius Simões, Juan David López-Ponce de León, Fabio Fernandes, Erika María Martínez-Carreño, Juan Pablo Costabel, Enrique A. Berrios-Bárcenas, Fabio Barroso, José Angel Cigarroa López, Rosa Ortega Reyes, Jaime Alvarez, Diego Pérez de Arenaza, Diana Buitrago, Constanza Otero, Claudenice Leite Bertoli de Souza, Juan Sebastián Molina Gómez, Guilherme Silva Julian

**Affiliations:** 1 Former Pfizer Global and Regional Medical Lead, Rare Disease, New York City, New York, United States of America; 2 CEPARM. Amyloidosis center. University Hospital, Federal University of Rio de Janeiro. Complexo hospitalar Américas – Vitória - Samaritano Barra, Rio de Janeiro, Brazil; 3 Department of Heart Failure and Heart Transplantation, Fundación Cardioinfantil-La Cardio, Bogotá, Colombia; 4 Medical School of Ribeirão Preto, University of São Paulo, São Paulo, Brazil; 5 Fundación Valle del Lili, Centro de Investigaciones Clínicas, Cali, Colombia; 6 Facultad de Ciencias de la Salud, Universidad Icesi, Cali, Colombia; 7 Departamento de Cardiología (Department of Cardiology), Fundación Valle del, Lili, Cali, Colombia; 8 Heart Institute, University of São Paulo, São Paulo, Brazil; 9 Department of Cardiology. Heart Failure, Clínica Iberoamerica, Barranquilla, Colombia; 10 Instituto Cardiovascular De Buenos Aires, Buenos Aires, Argentina; 11 Head of Cardiomyopathies Clinic - Instituto Nacional de Cardiología Ignacio Chávez, Mexico City, México; 12 Head of Neuromuscular Service at FLENI Foundation, Buenos Aires, Argentina; 13 Hospital de Cardiología Centro Médico Nacional Siglo XXI, Instituto Mexicano del Seguro Social, Ciudad de México, Mexico; 14 Unidad de cardiología, Hospital San Pablo de Coquimbo, Coquimbo, Chile; 15 Unidad de Intensivo Cardiovascular, Clínica BUPA Santiago, Santiago, Chile; 16 Head of Cardiovascular Imaging, Hospital Italiano de Buenos Aires, Buenos Aires, Argentina; 17 Real World Insights, IQVIA, Colombia, Bogotá; 18 Real World Insights, IQVIA, Chile, Santiago; 19 Pfizer, São Paulo, Brazil; 20 Pfizer, Bogotá, Colombia; Oswaldo Cruz Foundation, BRAZIL

## Abstract

**Background:**

Cardiac Amyloidosis (CA), specifically Transthyretin Amyloid Cardiomyopathy (ATTR-CM), is an under-recognized, progressive, and fatal disease. Comprehensive primary data describing awareness, attitudes, and barriers to treatment in Latin America (LATAM) are lacking, highlighting a gap between guidelines and real-world clinical practice.

**Methods:**

A cross-sectional, questionnaire-based observational study was conducted among 470 physicians actively involved in patient management across 8 LATAM countries (June-August 2023). The study assessed their awareness, attitudes, and barriers concerning ATTR-CM. Descriptive analysis was performed.

**Results:**

Limited knowledge of ATTR-CM was observed. Awareness of its pathophysiology was reported by 50.2%. Patients consulted 4–5 physicians before receiving a confirmed diagnosis. Cardiologists managed most day-to-day cases (76.2%). Transthyretin genetic testing was available to only 26.4%. Local treatment protocols relied on international guidelines (87.4%), with Real-World Data considered important by 60.6%. Patient cost and access to tafamidis were frequently reported barriers. Follow-up commonly relied on echocardiography, NT-pro BNP, NYHA classification, and ECG/Holter ECG.

**Conclusions:**

Difficulties in ATTR-CM diagnosis and management in LATAM were influenced by varying knowledge levels, gaps in diagnostic algorithms, and significant access barriers. These factors contribute to delayed diagnosis and management. Enhanced, tailored training strategies and collaborative efforts are necessary to improve early diagnosis, effective treatment, and ultimately, patient outcomes in the region.

## Introduction

Cardiac Amyloidosis (CA) is a rare disorder characterized by the accumulation of mis-folded proteins in cardiac tissue, leading to progressive cardiac dysfunction. Several types of amyloidosis exist; however, the most common forms affecting the heart are Transthyretin Amyloid Cardiomyopathy (ATTR-CM) and Amyloid Light chain (AL) or primary amyloidosis. ATTR-CM results from the deposition of transthyretin protein and can occur as a hereditary condition or as an acquired disorder associated with age-related changes [1].

ATTR-CM is a frequently overlooked, progressive, and potentially fatal disease. In its early stages, clinical symptoms may resemble hypertensive heart disease or hypertrophic cardiomyopathy [1]. Disease progression is unpredictable, as the underlying genotype influences both the likelihood and extent of cardiac involvement in hereditary variants. ATTR-CM is often misdiagnosed as other forms of cardiomyopathy, making its true prevalence difficult to estimate despite its severity. However, recent studies suggest that the prevalence of transthyretin amyloidosis may be higher than previously thought [2–4], particularly in specific patient sub-groups, such as older adults with aortic stenosis, or patients with left ventricular hypertrophy and Heart Failure (HF) with preserved ejection fraction [5].

ATTR-CM is a chronic, systemic, and potentially life-threatening condition that requires continuous, complex, and interdisciplinary diagnostic evaluation and follow-up. In Latin America, however, comprehensive studies based on primary data collection describing awareness, attitudes, and barriers related to the management of this disease are lacking. A recent survey involving cardiologists (65%) and general practitioners (17%), reported limited knowledge of CA among healthcare professionals (HCP) in the region. That study concluded that, due to the variable clinical presentation, overall survival is poor once advanced cardiac involvement occurs, and the effectiveness of emerging therapies is strongly dependent on timely diagnosis and access to treatment [6]. Increased awareness among HCP is therefore critical to ensure that amyloidosis is considered in the differential diagnosis and to improve patient outcomes [6].

In this context, the prevalence of ATTR-CM in Latin America may be slightly higher than in other regions [6–8]. Although the underlying reasons remain incompletely understood, certain mutations in the transthyretin gene [7], along with environmental or lifestyle factors, may contribute to this increased prevalence, particularly among older population [6]. Despite the limited data, ATTR-CM represents an important public health concern in Latin America [6], as early diagnosis and appropriate treatment are essential to improve outcomes and to better understand disease determinants in this population [2,6,9].

Consequently, a significant knowledge gap remains in Latin America regarding how real-world clinical practices align with current medical society recommendations [1,2,7,10–15]. Improving this understanding could directly benefit patients by informing the development of educational initiatives aimed at addressing unmet needs related to disease awareness, self-management, genetic counseling, and patient support resources.

To address this gap, the present study aims to assess awareness, attitudes, and barriers related to the diagnosis, treatment, and follow-up of ATTR-CM among HCP in Latin America. By examining real-world practices and perceived challenges in managing ATTR-CM, this study seeks to identify opportunities for targeted interventions, education, and improvement in clinical care.

## Materials and methods

This observational, cross-sectional study was conducted using a questionnaire-based approach to evaluate awareness, attitudes, and perceived barriers related to the diagnosis, treatment, and follow-up of ATTR-CM, including wild type ATTR-CM (ATTR-wt), hereditary ATTR-CM (ATTRv), and mixed phenotypes, among physicians in Latin America. This study is reported in accordance with the STROBE guidelines for cross-sectional studies.

The target population consisted of HCP specialized in Internal Medicine, Cardiology or Neurology, who were actively involved in the clinical management of patients with amyloidosis, HF, or HF with preserved ejection fraction. Eligible participants were physicians practicing in Argentina, Brazil, Chile, Colombia, Mexico, and selected Central America and Caribbean Countries (Costa Rica, Dominican Republic, and Panama – CENCA).

The questionnaire was developed ad hoc by a multidisciplinary panel of experts with experience in Cardiology, Neurology, Internal Medicine and amyloidosis care. The survey was designed to assess physicians’ sociodemographic characteristics, disease awareness, diagnostic approaches, treatment practices, and follow-up strategies related to ATTR-CM. Prior to full dissemination, the questionnaire was piloted among a small group of physicians’ representatives of the target population to assess clarity, relevance, and completeness of the items. Minor wording adjustments were made based on this feedback. The final questionnaire was administered in Spanish and Portuguese, according to participants’ country.

The survey was disseminated online using the Merphin platform, which ensured participant anonymity and data security. Physicians were invited to participate via e-mail from an existing IQVIA HCP database, using targeted invitations to relevant specialties and clinical areas. IQVIA managed physician invitations in accordance with applicable data protection regulations, and no personally identifiable information was shared with the authors.

Eligible physicians registered in the IQVIA database, residing in one of the participating countries and with at least one of the selected specialties, were invited via email to participate in the survey. Due to the survey dissemination strategy, a formal response rate could not be calculated. Only fully complete questionnaires were included in the analytical sample. Data collection took place between June 2023 and August 2023. All participants provided electronic written informed consent prior to accessing the questionnaire.

The questionnaire included sections on sociodemographic variables, awareness of ATTR-CM, diagnostic pathways, treatment practices, and follow-up strategies. Combined dimensions were created for knowledge of the disease and attitudes toward diagnosis. The full questionnaire and the anonymized database are available as Supplementary Files (S1 and S2).

The primary analysis was descriptive, summarizing participant characteristics and survey responses using measures of central tendency and dispersion (mean, standard deviation, median and range). Although no formal hypotheses were tested, exploratory inferential analyses were conducted to identify potential associations between variables. Bivariate and multivariate analyses were conducted using ANOVA for comparisons across groups. These analyses were considered exploratory and hypothesis-generating rather than confirmatory. Statistically significant correlations were identified with p < 0.05. All statistical analyses were performed using R software version 4.3.3.

### Ethical considerations

All participants provided electronic informed consent prior to participation. The survey was anonymous, and no sensitive personal data were collected.

Given that this was a non-interventional, anonymous survey conducted among HCP and did not involve patient data, formal approval from an institutional review board or independent ethics committee was not required in accordance with applicable local regulations. Nevertheless, the study was conducted following the Good Epidemiology Practices and the ethics principles outlined in the Declaration of Helsinki, including voluntary participation, confidentiality and minimization of potential risk to participants.

## Results

### Sociodemographic data & Practice setting

A total of 470 physicians were included in the analysis. Most respondents were male (66.6%), and cardiology was the most frequently reported primary medical specialty. The mean number of years of practice in the primary specialty was 14.4 years. The most common work settings were academic hospitals and private clinic/office, reported by 43.2% and 41.1% of participants, respectively. A substantial proportion of physicians reported involvement in at least one scientific organization (60.4%). Additional details are presented in Table 1.

**Table 1:** Sociodemographic environment of physicians included in the study.

	Argentina (N = 80)	Brazil (N = 120)	Chile (N = 50)	Colombia (N = 80)	Mexico (N = 90)	CENCA (N = 50)	Total (N = 470)
**Sex**							
Female	39 (48.8%)	38 (31.7%)	16 (32.0%)	18 (22.5%)	25 (27.8%)	21 (42.0%)	157 (33.4%)
Male	41 (51.2%)	82 (68.3%)	34 (68.0%)	62 (77.5%)	65 (72.2%)	29 (58.0%)	313 (66.6%)
**Primary medical specialty**							
Cardiology	50 (62.5%)	70 (58.3%)	40 (80.0%)	50 (62.5%)	70 (77.8%)	31 (62.0%)	311 (66.2%)
Internal Medicine	10 (12.5%)	20 (16.7%)	10 (20.0%)	30 (37.5%)	20 (22.2%)	19 (38.0%)	109 (23.2%)
Neurology	20 (25.0%)	30 (25.0%)	0 (0.0%)	0 (0.0%)	0 (0.0%)	0 (0.0%)	50 (10.6%)
**Years of practice in the primary specialty**							
Mean (SD)	18.1 (10.8)	15.9 (8.2)	13.9 (9.0)	8.6 (8.0)	14.7 (10.1)	14.0 (9.4)	14.4 (9.7)
Range	1.0 - 51.0	1.0 - 43.0	1.0 - 33.0	1.0 - 36.0	2.0 - 46.0	3.0 - 35.0	1.0 - 51.0
**Specific work setting**							
Academic hospital	35 (43.8%)	31 (25.8%)	25 (50.0%)	36 (45.0%)	47 (52.2%)	29 (58.0%)	203 (43.2%)
Low complexity hospital	1 (1.2%)	1 (0.8%)	1 (2.0%)	3 (3.8%)	3 (3.3%)	2 (4.0%)	11 (2.3%)
Non-academic, high-complexity hospital	10 (12.5%)	22 (18.3%)	7 (14.0%)	13 (16.2%)	9 (10.0%)	2 (4.0%)	63 (13.4%)
Private Clinic/Office	34 (42.5%)	66 (55.0%)	17 (34.0%)	28 (35.0%)	31 (34.4%)	17 (34.0%)	193 (41.1%)
**Scientific Organization**							
No	33 (41.2%)	110 (91.7%)	24 (48.0%)	32 (40.0%)	64 (71.1%)	21 (42.0%)	284 (60.4%)
Yes	47 (58.8%)	10 (8.3%)	26 (52.0%)	48 (60.0%)	26 (28.9%)	29 (58.0%)	186 (39.6%)

N = number of physicians; SD: standard deviation

### Knowledge and awareness

Regarding knowledge of ATTR-CM, 221 participants (47.0%) reported having a fair amount of knowledge, 41 (8.7%) reported having a great deal of knowledge, 32 (6.8%) had heard of the disease but knew nothing about it, 166 (35.3%) reported having only a little knowledge, and 10 (2.1%) had never heard of ATTR-CM.

Among physicians reporting low to moderate knowledge of ATTR-CM, 36.6% were cardiologists, 61.5% were internists, and 54.0% were neurologists. Most within this group did not belong to related scientific organizations (64.4%). The primary practice settings were private clinics/offices (43.4%) and academic hospitals (39.4%). During the previous 12 months, 51.0% reported managing no amyloidosis cases, and 42.3% reported managing up to 5 cases. In contrast, exposure to HF was higher: 38.9% reported managing between 50 and 250 HF patients, and for HF with preserved ejection fraction, 46.2% reported managing up to 100 patients.

At the regional level, the proportion of physicians reporting low to moderate knowledge of ATTR-CM was 56.2% in Argentina, 46.6% in Brazil, 44.0% in Chile, 29.9% in Colombia, 41.1% in Mexico, and 48.0% in CENCA.

In the bivariate analyses, no significant association was identified between years of practice in the specialty and disease knowledge (p = 0.93), despite a tendency toward higher years of experience among physicians reporting lower levels of knowledge. Similarly, multivariate analysis showed no statistically significant differences in knowledge when adjusting for sex, country, specialty, or work environment (p = 0.07, 0.3, 0.13, 0.58, respectively).

In terms of disease awareness (Table 2), 66.2% of respondents agreed or strongly agreed with the statement “I am aware of the pathophysiology of ATTR-CM”. Physicians reported encountering suspected ATTR-CM cases monthly, semi-annually, or less than annually (28.3%, 24.3%, and 23.6%, respectively). Patients were reported to consult an average of 4–5 physicians prior to diagnosis, and cardiologists were identified as being primarily responsible for day-to-day patient management (76.2%).

**Table 2:** Awareness of the disease.

	Argentina (N = 80)	Brazil (N = 120)	Chile (N = 50)	Colombia (N = 80)	Mexico (N = 90)	CENCA (N = 50)	Total (N = 470)
**To be aware of the pathophysiology of ATTR-CM**							
Strongly Agree	7 (8.8%)	18 (15.0%)	9 (18.0%)	15 (18.8%)	16 (17.8%)	10 (20.0%)	75 (16.0%)
Agree	36 (45.0%)	60 (50.0%)	25 (50.0%)	42 (52.5%)	48 (53.3%)	25 (50.0%)	236 (50.2%)
Neutral	30 (37.5%)	22 (18.3%)	8 (16.0%)	13 (16.2%)	14 (15.6%)	11 (22.0%)	98 (20.9%)
Disagree	2 (2.5%)	12 (10.0%)	5 (10.0%)	5 (6.2%)	3 (3.3%)	1 (2.0%)	28 (6.0%)
Strongly Disagree	5 (6.2%)	8 (6.7%)	3 (6.0%)	5 (6.2%)	9 (10.0%)	3 (6.0%)	33 (7.0%)
**Frequency of suspicion of ATTR-CM**							
Weekly	4 (5.0%)	14 (11.7%)	1 (2.0%)	10 (12.5%)	4 (4.4%)	4 (8.0%)	37 (7.9%)
Monthly	15 (18.8%)	36 (30.0%)	17 (34.0%)	33 (41.2%)	25 (27.8%)	7 (14.0%)	133 (28.3%)
Semi-annually	22 (27.5%)	23 (19.2%)	9 (18.0%)	17 (21.2%)	26 (28.9%)	17 (34.0%)	114 (24.3%)
Less frequently than annually	27 (33.8%)	20 (16.7%)	14 (28.0%)	9 (11.2%)	23 (25.6%)	18 (36.0%)	111 (23.6%)
Annually	12 (15.0%)	27 (22.5%)	9 (18.0%)	11 (13.8%)	12 (13.3%)	4 (8.0%)	75 (16.0%)
**Physicians consulted before receiving a confirmed diagnosis of ATTR-CM**							
0	3 (3.8%)	7 (5.8%)	3 (6.0%)	6 (7.5%)	6 (6.7%)	8 (16.0%)	33 (7.0%)
1-3	31 (38.8%)	36 (30.0%)	18 (36.0%)	17 (21.2%)	38 (42.2%)	18 (36.0%)	158 (33.6%)
4-5	33 (41.2%)	52 (43.3%)	17 (34.0%)	32 (40.0%)	34 (37.8%)	20 (40.0%)	188 (40.0%)
6-8	6 (7.5%)	15 (12.5%)	6 (12.0%)	15 (18.8%)	6 (6.7%)	3 (6.0%)	51 (10.9%)
9-10	1 (1.2%)	1 (0.8%)	1 (2.0%)	2 (2.5%)	3 (3.3%)	0 (0.0%)	8 (1.7%)
>10	6 (7.5%)	9 (7.5%)	5 (10.0%)	8 (10.0%)	3 (3.3%)	1 (2.0%)	32 (6.8%)
**Primary (day-to-day) management (treatment, follow up) responsibility of patients with ATTR-CMA**							
A cardiologist	70 (87.5%)	96 (80.0%)	38 (76.0%)	60 (75.0%)	58 (64.4%)	36 (72.0%)	358 (76.2%)
A family physician	0 (0.0%)	2 (1.7%)	1 (2.0%)	1 (1.2%)	3 (3.3%)	0 (0.0%)	7 (1.5%)
A general practitioner	1 (1.2%)	0 (0.0%)	2 (4.0%)	0 (0.0%)	2 (2.2%)	1 (2.0%)	6 (1.3%)
A hematologist	2 (2.5%)	6 (5.0%)	1 (2.0%)	6 (7.5%)	9 (10.0%)	3 (6.0%)	27 (5.7%)
A neurologist	1 (1.2%)	5 (4.2%)	0 (0.0%)	0 (0.0%)	0 (0.0%)	0 (0.0%)	6 (1.3%)
An internal medicine physician	4 (5.0%)	6 (5.0%)	3 (6.0%)	10 (12.5%)	6 (6.7%)	6 (12.0%)	35 (7.4%)
I am unsure	2 (2.5%)	5 (4.2%)	5 (10.0%)	3 (3.8%)	12 (13.3%)	4 (8.0%)	31 (6.6%)

N = number of physicians

**Fig 1:**
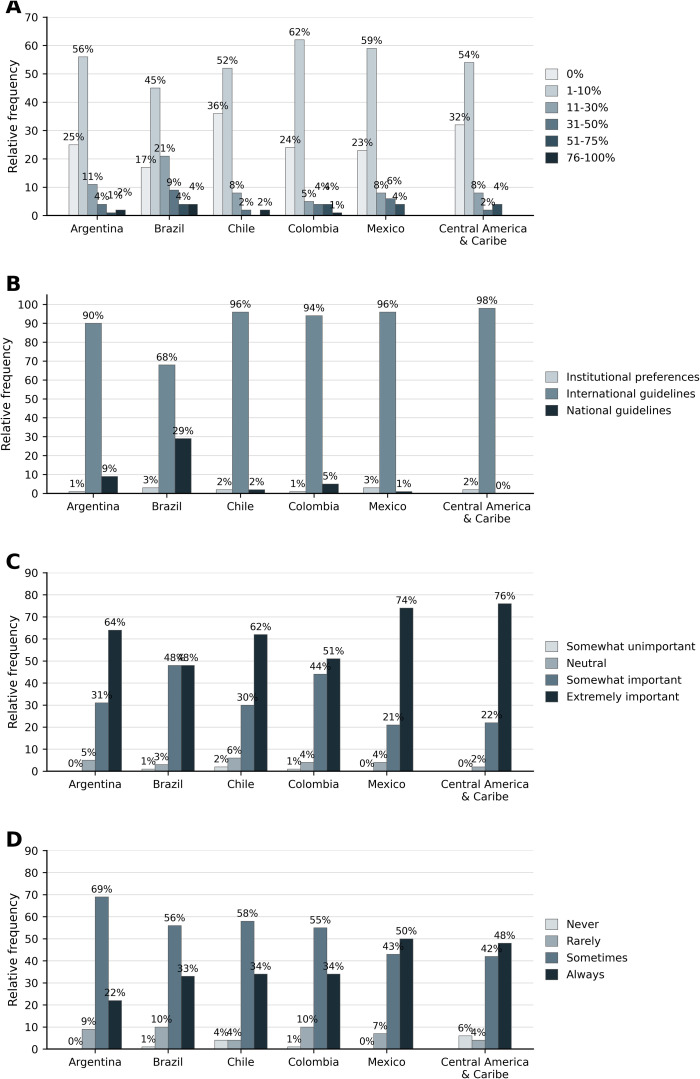
Distribution of responses provided by physicians related to awareness and knowledge. **A.** Percentage of patients ever encountered with Transthyretin Amyloid Cardiomyopathy; **B.** Treatment guidelines: If local treatment protocols were conducted, what would they be based on?; **C.** Importance of Real-World Data when making treatment decisions; **D.** The patients feel fully informed and understand their diseases, treatment, and prognosis when it is explained by the physician.

Overall clinical exposure to ATTR-CM was low. Fig 1 shows that 24.3% of respondents reported that ATTR-CM accounted for 0% of their patients, while 54.3% reported that it represented between 1% and 10% of their caseload. Only 1.9% of respondents reported ATTR-CM accounting for 76% to 100% of their patients. Similar distributions were observed across ATTR-CM subtypes.

Among physicians reporting low awareness of ATTR-CM (those who strongly disagreed or disagreed with the awareness statement), 9.9% were cardiologists, 23.8% were internists, and 8.0% were neurologists. Most did not belong to a related scientific organization (67.4%). Regarding patient management, 57.4% reported managing no amyloidosis patients in the previous 12 months, while 39.3% reported managing up to 5. Practice settings included private clinics/offices (42.6%), academic hospitals (41.0%), and non-academic high-complexity hospitals (16.4%). Regional distribution included Argentina (8.7%), Brazil (16.7%), Chile (16.0%), Colombia (12.4%), Mexico (13.3%), and CENCA (8.0%).

### Diagnostic tools and practices

Reported availability of diagnostic imaging varied. Bone scintigraphy was available to 65.1% of respondents, while Cardiac MRI (cMRI) was available to 71.9%. Echocardiogram with and without strain were available to 81.9% and 95.7% of respondents, respectively. Only 26.4% reported access to transthyretin genetic testing. Biomarker availability included NT-pro BNP (83.6%), serum/urine protein electrophoresis (84.7%), free-light chain assay (FLC - 55.7%), and troponin T/I (95.1%).

Reported utilization frequency over the preceding 12 months varied considerably. Bone scintigraphy was not performed by 34.0% of respondents, while 34.3% performed fewer than 5 scans. Cardiac amyloid radionuclide imaging was performed fewer than 5 times by 30.2% of respondents and more than 45 times by 6.0%. Echocardiogram with strain was not performed by 17.4% of respondents, while 21.9% performed it fewer than 5 times and 18.1% performed more than 45 examinations. Similar variability was observed for echocardiogram without strain, where 14.9% of respondents did not perform the exam, 20.2% of respondents performed fewer than 5 times, and 29.6% performed more than 45 times.

Transthyretin genetic testing was not performed by 40.2% of respondents, while 39.4% performed fewer than 5 tests, and 1.9% performed more than 45. NT-pro BNP testing was not performed by 11.9%, while 21.9% performed fewer than 5 and 28.1% performed more than 45 tests.

Bone scintigraphy was rated a high priority diagnostic tool by 31.9% of respondents, medium priority by 39.1%, and low priority by 28.9%. Transthyretin genetic testing was rated as high priority by 74.5%, NT-proBNP by 27.9%, serum/urine protein electrophoresis by 40.6%, FLC by 48.7%, Troponin T/I by 21.7%. Non-cardiac tissue biopsy and cardiac biopsy were rated as high priority by 32.3% and 44.0% of respondents, respectively. Mass spectrometry was rated as high priority by 38.1% of respondents and immunohistochemistry by 56.2%. Quantitative cardiac SPECT/PET was rated as high priority by 43.2% of respondents.

Multivariate analyses showed that most responses clustered around “agree” or “strongly agree” regarding attitudes toward diagnosis (Table 3). No association was found with years of practice. Statistically significant differences were observed between Mexico and Argentina (p = 0.02), and between Mexico and Brazil (p < 0.01) and higher attitude scores were observed in Mexico. Cardiologists showed higher scores than internists (p = 0.02). No significant differences were observed by sex (p = 0.8), or work environments (p = 0.13).

**Table 3:** Attitudes and barriers toward the diagnosis of the disease.

	Argentina (N = 80)	Brazil (N = 120)	Chile (N = 50)	Colombia (N = 80)	Mexico (N = 90)	CENCA (N = 50)	Total (N = 470)
**To be aware of the risk factors for ATTR-CM**							
Strongly Agree	7 (8.8%)	16 (13.3%)	6 (12.0%)	13 (16.2%)	9 (10.0%)	6 (12.0%)	57 (12.1%)
Agree	30 (37.5%)	58 (48.3%)	23 (46.0%)	38 (47.5%)	54 (60.0%)	26 (52.0%)	229 (48.7%)
Neutral	35 (43.8%)	28 (23.3%)	13 (26.0%)	20 (25.0%)	19 (21.1%)	12 (24.0%)	127 (27.0%)
Disagree	4 (5.0%)	12 (10.0%)	5 (10.0%)	2 (2.5%)	5 (5.6%)	2 (4.0%)	30 (6.4%)
Strongly Disagree	4 (5.0%)	6 (5.0%)	3 (6.0%)	7 (8.8%)	3 (3.3%)	4 (8.0%)	27 (5.7%)
**To be aware of the risk symptoms of ATTR-CM**							
Strongly Agree	15 (18.8%)	28 (23.3%)	11 (22.0%)	23 (28.8%)	18 (20.0%)	10 (20.0%)	105 (22.3%)
Agree	46 (57.5%)	71 (59.2%)	27 (54.0%)	43 (53.8%)	59 (65.6%)	30 (60.0%)	276 (58.7%)
Neutral	16 (20.0%)	13 (10.8%)	7 (14.0%)	8 (10.0%)	8 (8.9%)	9 (18.0%)	61 (13.0%)
Disagree	1 (1.2%)	4 (3.3%)	2 (4.0%)	2 (2.5%)	2 (2.2%)	0 (0.0%)	11 (2.3%)
Strongly Disagree	2 (2.5%)	4 (3.3%)	3 (6.0%)	4 (5.0%)	3 (3.3%)	1 (2.0%)	17 (3.6%)
**To agree with the fact that timely diagnosis of ATTR-CM is uncommon**							
Strongly Agree	18 (22.5%)	17 (14.2%)	23 (46.0%)	33 (41.2%)	39 (43.3%)	24 (48.0%)	154 (32.8%)
Agree	43 (53.8%)	67 (55.8%)	20 (40.0%)	42 (52.5%)	42 (46.7%)	19 (38.0%)	233 (49.6%)
Neutral	12 (15.0%)	17 (14.2%)	5 (10.0%)	4 (5.0%)	5 (5.6%)	6 (12.0%)	49 (10.4%)
Disagree	5 (6.2%)	18 (15.0%)	1 (2.0%)	1 (1.2%)	3 (3.3%)	1 (2.0%)	29 (6.2%)
Strongly Disagree	2 (2.5%)	1 (0.8%)	1 (2.0%)	0 (0.0%)	1 (1.1%)	0 (0.0%)	5 (1.1%)
**To agree with the fact that timely diagnosis of ATTR-CM delays appropriate management**							
Strongly Agree	40 (50.0%)	65 (54.2%)	28 (56.0%)	49 (61.2%)	56 (62.2%)	27 (54.0%)	265 (56.4%)
Agree	36 (45.0%)	53 (44.2%)	19 (38.0%)	29 (36.2%)	28 (31.1%)	21 (42.0%)	186 (39.6%)
Neutral	3 (3.8%)	1 (0.8%)	3 (6.0%)	2 (2.5%)	5 (5.6%)	2 (4.0%)	16 (3.4%)
Disagree	1 (1.2%)	0 (0.0%)	0 (0.0%)	0 (0.0%)	1 (1.1%)	0 (0.0%)	2 (0.4%)
Strongly Disagree	0 (0.0%)	1 (0.8%)	0 (0.0%)	0 (0.0%)	0 (0.0%)	0 (0.0%)	1 (0.2%)

N = number of physicians

Statistically significant differences in attitudes toward diagnosis were observed in CENCA region, Mexico and Colombia compared with Argentina (p = 0.01, 0.02 and 0.04, respectively) with mean attitude score nearly twice as high in these regions. No statistically significant differences were observed by sex, specialty or years of practice after adjustment for other variables.

### Barriers to healthcare

Physicians emphasized the need for improved awareness, with 64.0% strongly agreeing that cardiologists require better education, and 69.4% strongly agreeing that other practitioners also require improved knowledge. Barriers to access diagnostic tools and biomarkers were strongly acknowledged, although fewer respondents reported barriers to risk-stratification tools (39.4%).

Strong agreement was also reported regarding the need for improved training in evaluating extracardiac manifestations (54.9%), identifying red flags (56.8%), understanding age-related disease manifestations (53.2%), and genotype-phenotype relationships (54.0%).

Regarding access barriers, 54.0% of physicians agreed that although genetic testing is available, awareness of its importance is limited. Barriers to outpatient and inpatient care were reported by 55.1% and 56.4% of respondents, respectively. Substantial barriers to genetic testing (44.7% and 46.0% of respondents agreed or strongly agreed, respectively) and counseling access were also reported (43.6% and 47.4% of respondents agree or strongly agreed, respectively).

Approximately half of respondents (51.3%) reported transthyretin genetic testing is available only in major centers, while 35.7% reported access through industry-sponsored support (Table 4).

**Table 4:** Availability of genetic testing.

	Argentina (N = 80)	Brazil (N = 120)	Chile (N = 50)	Colombia (N = 80)	Mexico (N = 90)	CENCA (N = 50)	Total (N = 470)
**Transthyretin Amyloidosis genetic testing availability in the corresponding country**							
It is not available	4 (5.0%)	10 (8.3%)	6 (12.0%)	1 (1.2%)	7 (7.8%)	17 (34.0%)	45 (9.6%)
It is available through industry-sponsored support	35 (43.8%)	28 (23.3%)	19 (38.0%)	47 (58.8%)	24 (26.7%)	15 (30.0%)	168 (35.7%)
It is available only in major centers	37 (46.2%)	79 (65.8%)	24 (48.0%)	29 (36.2%)	56 (62.2%)	16 (32.0%)	241 (51.3%)
It is widely available	4 (5.0%)	3 (2.5%)	1 (2.0%)	3 (3.8%)	3 (3.3%)	2 (4.0%)	16 (3.4%)

N = number of physicians

The highest proportion of responses indicated a moderate level of barriers to diagnosis, with most scores clustering around 0.6 score on a scale ranging from 0 (no barriers) to 1 (a high number of barriers). No correlation was observed between barrier scores and the average years of specialty practice.

In contrast, barrier scores varied significantly across countries (p < 0.01), with Colombia reporting the fewest barriers to diagnosis. Barriers to diagnosis were more frequently reported in low-complexity hospitals compared with other practice settings (p < 0.01). No statistically significant differences in barriers to diagnosis were observed across medical specialties (p = 0.05).

### Treatment

When physicians were asked if they agreed with the statement “ATTR-CM is an untreatable disease”, 49.3% disagreed or strongly disagreed (Table 5).

**Table 5:** ATTR-CM is an untreatable disease.

	Argentina (N = 80)	Brazil (N = 120)	Chile (N = 50)	Colombia (N = 80)	Mexico (N = 90)	CENCA (N = 50)	Total (N = 470)
**ATTR-CM is an untreatable disease**							
Strongly Agree	9 (11.2%)	0 (0.0%)	4 (8.0%)	7 (8.8%)	12 (13.3%)	6 (12.0%)	38 (8.1%)
Agree	24 (30.0%)	4 (3.3%)	16 (32.0%)	24 (30.0%)	31 (34.4%)	19 (38.0%)	118 (25.1%)
Neutral	20 (25.0%)	15 (12.5%)	9 (18.0%)	18 (22.5%)	14 (15.6%)	6 (12.0%)	82 (17.4%)
Disagree	26 (32.5%)	69 (57.5%)	18 (36.0%)	25 (31.2%)	25 (27.8%)	13 (26.0%)	176 (37.4%)
Strongly Disagree	1 (1.2%)	32 (26.7%)	3 (6.0%)	6 (7.5%)	8 (8.9%)	6 (12.0%)	56 (11.9%)

N = number of physicians

Regarding treatment choice and preferences, Fig 1 summarizes the factors on which local treatment protocols are based. Among respondents, 87.4% indicated that international guidelines were the primary basis for local protocols, whereas 10.2% reported relying on national guidelines and only 2.3% cited institutional preferences. This distribution highlights a strong reliance on internationally recognized guidelines to guide treatment decisions.

Fig 1 also illustrates the perceived importance of Real-World Data (RWD) in informing treatment decisions. Overall, 60.6% of participants considered RWD to be very important, while 34.7% rated it as somewhat important. A smaller proportion reported neutral views (4.0%), and only 0.6% considered RWD to be somewhat unimportant. These findings underscore the prominent role attributed to real-world evidence in clinical decision-making among respondents.

The most influential factors guiding the prescription of a specific therapy were disease progression (19.6%) and the availability of a given treatment in physicians’ practice (19.5%). Patients’ ability to pay also played a relevant role, accounting for 13.6% of responses. Factors identified as potentially increasing the use of specific treatments included an overall reduction in treatment costs (18.9%), greater availability of treatments in a specific setting (18.0%), additional physician training (17.2%), improved patient education tools (14.3%), and inclusion of treatments in local or institutional guidelines (14.1%).

Cost-related barriers to treatment access were widely acknowledged. A total of 60.4% of respondents strongly agreed, and 33.0% agreed that patients face significant financial barriers to care. Similarly, barriers to accessing tafamidis were reported, with 58.7% strongly agreed, and 31.9% agreeing that access to this therapy remains challenging.

### Follow-up and patient’s perspective

When physicians were asked which tools they routinely use as part of patients follow-up, the most frequently reported were echocardiographic measures of left ventricular wall thickness/mass (14.0%), NT-pro BNP (12.9%), echocardiographic assessment of diastolic function (12.1%), echocardiographic assessment of systolic function (12.0%), NYHA functional class evaluation (12.0%) and electrocardiography or Holter monitoring (11.5%).

Regarding patient self-empowerment in managing their disease, physicians were asked about the information sources on which patients most commonly rely to improve their understanding. The most frequently cited sources were internet websites (38.9%), newspaper or magazine articles (15.7%), nurses (12.2%), patient support groups (12.1%), and family members or friends (11.7%).

With respect to patients’ perceived understanding of their disease, treatment, and prognosis (Fig 1), 36.4% of HCP reported that they always feel their patients are fully informed after these aspects are explained. In contrast, 54.3% indicated that patients are only sometimes fully informed, 7.9% reported that patients are rarely fully informed, and 1.5% stated that patients are never fully informed. These findings emphasize the need for ongoing efforts to strengthen patient education and communication throughout the disease course.

### Differences between specialties

Regarding knowledge of ATTR-CM across specialties, a fair level of awareness was reported among all three groups, with cardiologists showing the highest proportion (52.7%), followed by neurologists (42.0%) and internists (41.3%). Among cardiology specialists, most respondents (53.4%) reported having a fair amount of knowledge of ATTR-CM, whereas neurologists exhibited the lowest proportion reporting this level of knowledge (36.0%).

Overall, 71.1% of cardiologists agreed or strongly agreed that they understood the pathophysiology of ATTR-CM, compared to 62.0% of neurologists and 54.2% of internists. Among neurologists, 14.0% reported suspecting the disease on a weekly basis and 20.0% on a monthly basis. Cardiologists showed a similar pattern, with 8.0% reporting weekly suspicion and 32.5% reporting monthly suspicion. In contrast, internists reported the lowest levels of suspicion, with 4.6% suspecting ATTR-CM weekly and 20.2% monthly.

Across all specialties, there was strong agreement that timely diagnosis is crucial for appropriate disease management. With respect to barriers related to access to risk-stratification tools, a higher proportion of neurologists (82.0%) and internists (79.9%) agreed or strongly agreed that such barriers exist, compared to cardiologists (70.4%). In addition, there was broad consensus across specialties regarding the need to improve knowledge and training for identifying disease red flags. Agreement on this need was highest among neurologists (96.0%), followed by cardiologists (94.5%) and internists (92.8%).

## Discussion

This study provides a comprehensive overview of HCP self-reported awareness and diagnostic practices related to ATTR-CM across several Latin American countries. Although physicians from Argentina, Chile, and Mexico reported higher awareness of certain aspects of the disease, substantial gaps persist in understanding key elements such as genotype-phenotype interactions, and clinical staging systems.

Across the region, physicians reported variable levels of awareness with ATTR-CM pathophysiology and risk factors, with relatively higher self-reported awareness in Brazil and Mexico (58.7%). However, these findings should be interpreted with caution, as a substantial proportion of respondents across all countries reported limited clinical exposure, with more than half of them indicating that they had not managed any patients with ATTR-CM in the previous year. This suggests that despite perceived familiarity, under-recognition and underdiagnosis of ATTR-CM likely persists in routine clinical practice. These findings contrast with earlier reports by Mut et al. [6], where only 33% of clinicians reported adequate knowledge and 65% had not requested a single nuclear scan in the previous year, but still highlight ongoing diagnostic challenges in the region.

This gap is particularly relevant considering findings by AbouEzzedine et al [16], who demonstrated that systematic screening among patients with HF with preserved ejection fraction and increased wall thickness resulted in a nearly five-fold increase in ATTR-CM detection. Together, these observations underscore the importance of maintaining a high index of clinical suspicion and implementing structured diagnostic approaches to facilitate earlier identification of the disease.

Although NT-pro BNP was widely available across participating countries (83.6%), its inconsistent use may reflect heterogeneity in clinical protocols or limited awareness of its prognostic role in ATTR-CM. Garcia-Pavia et al. [12] emphasized the value of NT-pro BNP as a key biomarker for monitoring disease progression, recommending a 30% increase as a marker of clinical deterioration. Our findings suggest that despite availability, standardized use of NT-pro BNP and other biomarkers such as troponin or FLC remains limited, which may contribute to delayed diagnosis or misclassification of disease severity.

While access to echocardiography and cardiac MRI was generally reported, more advanced diagnostic tools, including genetic testing and advanced imaging techniques, were unevenly available. This aligns with prior reports from Mut et al., who described underutilization of nuclear imaging despite its widespread availability in the region [6]. Similarly, Dower et al. [17] highlighted the clinical diagnostic value of bone scintigraphy pyrophosphate tracers for early diagnosis and treatment planning. In our survey, however, only 31.9% of respondents considered bone scintigraphy a high-priority diagnostic tool, suggesting a disconnect between established evidence and perceived clinical utility. This gap may contribute to delays in diagnosis due to unsatisfactory diagnostic pathways and consequently delay treatment initiation.

Transthyretin genetic testing plays a pivotal role in confirming ATTRv and distinguishing it from ATTR-wt [1,18]. Beyond diagnostic confirmation, genetic testing enables familial screening and supports early identification of at-risk relatives, allowing appropriate surveillance and counseling. Genetic counseling based on test results provides critical support for patients and families, helping them understand inheritance patterns, disease implications, and reproductive options. Despite this importance, access to genetic testing remained limited among respondents, reinforcing the need to strengthen referral networks and access to specialized services. On the other hand, the low prioritization of bone scintigraphy is likewise concerning, particularly given its central role in contemporary non-invasive diagnosis algorithms when combined with laboratory testing to exclude AL amyloidosis, and can often obviate the need for endomyocardial biopsy [11,15].

The limited use of serum/urine protein electrophoresis and FLC assays further complicate accurate differentiation between ATTR-CM and AL amyloidosis, conditions that require distinct management strategies. As emphasized by Adams et al. [18], incorporating these assays into diagnostic algorithms is essential, particularly in regions where clinical suspicion may be low. Our findings reinforce this need, as only 26.4% of respondents reported access to transthyretin genetic testing, and many acknowledged barriers to genetic counseling and follow-up care for asymptomatic carriers.

Access to disease-modifying therapy also remains a major challenge. Cost-related barriers to tafamidis were reported across the region by the majority of the respondents (90.6%), despite strong evidence presented by Elliott et al. [19], in the “Tafamidis in Transthyretin Cardiomyopathy Clinical Trial”, demonstrating a 41% reduction in all-cause mortality with early tafamidis initiation, particularly with early initiation in patients with NYHA functional class I–II. This highlights a disconnect between robust clinical evidence and real-world access to therapy, particularly in lower-resource healthcare settings.

Beyond clinical outcomes, ATTR-CM also imposes a substantial burden on healthcare systems. Lauppe et al. [20] demonstrated that patients with ATTR-CM had nearly twice as many outpatient visits and 50% more hospitalizations in the year following diagnosis compared with patients with HF without ATTR-CM. Notably, increased healthcare resource utilization was already evident up to three years prior to diagnosis, suggesting multiple missed opportunities for earlier disease recognition. In this context, the limited awareness, inconsistent diagnostic practices, and restricted access to key diagnostic tools identified in our study further underscore the need for structured screening strategies and targeted education initiatives aimed at reducing diagnostic delays. In the absence of widely implemented local guidelines, the development and dissemination of standardized regional or national recommendations should be encouraged [11,15].

Overall, these findings point to actionable gaps that may inform targeted educational interventions, improved referral pathways, and health system-level strategies to enhance ATTR-CM recognition and management in Latin America. While the inclusion of multiple countries, many of them key regional healthcare leaders, enhances the relevance of the study, the results should be interpreted with caution when extrapolating to countries not represented in the sample. Within this context, efforts to expand access to transthyretin genetic testing, bone scintigraphy, and disease-modifying therapies such as tafamidis may contribute to earlier diagnosis, improved clinical outcomes, and a potential reduction in healthcare system burden in the region.

### Limitations

This study is subject to limitations inherent to survey-based, cross-sectional designs. First, the use of self-reported data may introduce biases such as social desirability and recall bias, as reported knowledge, attitudes, and clinical practices were not independently verified. In addition, the voluntary nature of participation may have led to self-selection bias, potentially resulting in an overrepresentation of physicians with greater interest in ATTR-CM awareness.

Although physicians from multiple specialties and several Latin American countries were included, the sample may not fully represent all physicians involved in the management of HF or related conditions in the region. In addition, the survey dissemination strategy did not allow for the calculation of a formal response rate, limiting the assessment of potential non-response bias.

Misinterpretation of survey questions is also possible despite the use of a structured and piloted questionnaire. Additionally, where composite scores were created to summarize knowledge and diagnostic attitudes, these measures were exploratory in nature, and the questionnaire did not undergo formal psychometric validation.

The cross-sectional design of the study captures physicians’ perception at a single point in time and does not allow assessment of temporal trends or causal relationships. Accordingly, these findings provide a valuable snapshot of current practices and may inform future longitudinal or intervention research to explore gaps in awareness and management of ATTR-CM.

## Conclusions

In conclusion, this multi-country survey provides a descriptive overview of physician-reported awareness, diagnostic practices, and perceived barriers related to ATTR-CM across Latin America. The findings illustrate substantial variability in familiarity with the disease, clinical exposure, and access to diagnostic resources, underscoring ongoing challenges in disease recognition and diagnosis. Although cardiologists play a central role in ATTR-CM management, the low number of reported patient encounters and the lack of standardized diagnostic pathways highlight important gaps in clinical preparedness across settings.

The results underscore the relevance of advanced imaging modalities and transthyretin genetic testing in ATTR-CM diagnosis, while also revealing significant opportunities to improve their consistent use and accessibility. The absence of harmonized diagnostic approaches suggests a need for structured diagnostic algorithms and referral pathways that may support earlier identification, appropriate follow up, and patient education.

Given the heterogeneity in self-reported knowledge levels and clinical practices across countries and healthcare settings, targeted educational initiatives tailored to local contexts may help address identified gaps. Collaborative efforts involving medical societies, healthcare institutions, and policymakers could inform region-specific strategies to improve ATTR-CM recognition, diagnostic workflow, and access to disease-modifying therapies. While findings should be interpreted cautiously given the descriptive and self-reported nature of the data, they provide a valuable foundation for future educational, organizational, and health system-level interventions aimed at improving ATTR-CM care in the region.

## Supporting information

S1 File Manuscript data. (XLSX)

S2 File Manuscript questionnaire. (DOCX)
