# Supplementary material for: Awareness, attitudes, and barriers toward Transthyretin Amyloid Cardiomyopathy in Latin America: A questionnaire-based cross-sectional study
Source: PLoS One. 2026 Jun 26;21(6):e0351224. doi: 10.1371/journal.pone.0351224 (PMC13309013; doi:10.1371/journal.pone.0351224)
Supplement: S2 File — (DOCX) [file pone.0351224.s002.docx]

1. **SCREENING, SOCIODEMOGRAPHIC AND PRACTICE SETTING**
2. Are you female or male?
   - Male
   - Female
3. Which country do you practice medicine in?
   - Argentina
   - Brazil
   - Chile
   - Colombia
   - México
   - Costa Rica
   - Dominican Republic
   - Panama
4. Which is your primary medical specialty?
   - Internal Medicine
   - Cardiology
   - Neurology
   - None of the above
5. Are you a practicing physician actively involved in the day-to-day management of patients of your primary specialty?

- Yes
- No

1. For how many years have you been practicing in your primary specialty? ________
2. Which of the following best describes your primary work setting?
   - Academic Hospital
   - Non-academic, high-complexity Hospital
   - Low complexity Hospital
   - Private Clinic/Office
3. Are you employed by a pharmaceutical company in any way, other than participating in clinical trials conducted by the company and/or advisory boards with contract?
   - Yes
   - No
4. Are you part of a Scientific Organization
   - Yes
   - No
   1. Please indicate below, by selecting the appropriate boxes, which organizations you are a part of:
   - Academia Brasileira de Neurología
   - Sociedade Brasileira de Cardiología
   - Academia Mexicana de Neurología
   - Sociedad Mexicana de Cardiología
   - Sociedad Neurológica Argentina
   - Sociedad Argentina de Cardiología
   - Sociedad de Neurología, Psiquiatría y Neurocirugía de Chile
   - Sociedad Chilena de Cardiología y Cirugía Cardiovascular
   - Asociación Colombiana de Neurología o Sociedad Colombiana de Cardiología y Cirugía Cardiovascular
   - Asociación Costarricense de Ciencias Neurologicas
   - Asociación Costarricense de Cardiología o Asociación Panameña de Neurología
   - Sociedad Panameña de Cardiología
   - Sociedad Dominicana de Neurologia y Neurocirugía
   - Sociedad Dominicana de Cardiologia
   - Sociedad Brasileira de Medicina Nuclear
   - American Society of Nuclear Cardiology (ASNC)
   - International Society of Internal Medicine (ISIM)
   - American College of Cardiology (ACC)
   - European Society of Cardiology (ESC)
   - World Heart Federation (WHF)
   - World Federation of Neurology (WFN)
   - American Neurological Association (ANA)
   - International Headache Society (IHS)
   - Heart Failure Society of America (HFSA)
   - Peripheral Nerve Society (PNS)
   - World Hypertension League (WHL)
   - Other (open field)
5. Please indicate below, by selecting the appropriate boxes, if you are personally involved in the care of patients with:
   - Amyloidosis
   - Heart failure
   - Heart failure with preserved ejection fraction
6. In the last 12 months, how many patients did you personally manage (please count each patient once, not the total number of visits) for the following conditions?
   1. Amyloidosis
   - Up to 5 patients
   - 5-20 patients
   - >20 patients
   1. Heart failure
   - Up to 50 patients
   - 50-250 patients
   - >250 patients
   1. Heart failure with preserved ejection fraction
   - Up to 100 patients
   - 100-500 patients
   - >500 patients
7. **AWARENESS OF THE DISEASE**
8. How much, if anything, would you say you know about Transthyretin Amyloidosis
   - A great deal
   - A fair amount
   - Just a little
   - Heard of, know nothing about it
   - Never heard of it
9. How much, if anything, would you say you know about Transthyretin Amyloid Cardiomyopathy?
   - A great deal
   - A fair amount
   - Just a little
   - Heard of, know nothing about it
   - Never heard of it
10. To what extent do you agree with each of the following statements about your current knowledge of Transthyretin Amyloid Cardiomyopathy?
    1. I am aware of the pathophysiology of Transthyretin Amyloid Cardiomyopathy
    - Strongly Disagree
    - Disagree
    - Neutral
    - Agree
    - Strongly Agree
11. Please indicate below, the percentage of patients you encounter with confirmed diagnosis of Transthyretin Amyloidosis according to its classification
    1. Transthyretin Amyloidosis Cardiomyopathy
    - 0%
    - 1% - 10%
    - 11% - 30%
    - 31% - 50%
    - 51% - 75%
    - 76% - 100%
    1. Wild-type Transthyretin Amyloidosis
    - 0%
    - 1% - 10%
    - 11% - 30%
    - 31% - 50%
    - 51% - 75%
    - 76% - 100%
    1. Hereditary Transthyretin Amyloidosis
    - 0%
    - 1% - 10%
    - 11% - 30%
    - 31% - 50%
    - 51% - 75%
    - 76% - 100%
    1. Transthyretin Amyloidosis Polyneuropathy
    - 0%
    - 1% - 10%
    - 11% - 30%
    - 31% - 50%
    - 51% - 75%
    - 76% - 100%
    1. Mixed phenotype Transthyretin Amyloidosis
    - 0%
    - 1% - 10%
    - 11% - 30%
    - 31% - 50%
    - 51% - 75%
    - 76% - 100%
12. How often do you encounter a patient with suspected Transthyretin Amyloid Cardiomyopathy?
    - Daily
    - Weekly
    - Monthly
    - Semi-Annually
    - Annually
    - Less frequently than annually
13. How many physicians the patients have consulted before receiving a confirmed diagnosis of Transthyretin Amyloid Cardiomyopathy
    - 0
    - 1-3
    - 4-5
    - 6-8
    - 8-10
    - >10
14. Have you ever been personally involved in the care of patients with Transthyretin Amyloid Cardiomyopathy?
    - Yes
    - No
15. Who provides the primary (day-to-day) management (treatment, follow up) of your patients that are diagnosed with Transthyretin Amyloid Cardiomyopathy
    - A general practitioner
    - A family physician
    - An internal medicine physician
    - A cardiologist
    - A neurologist
    - A hematologist
    - I am unsure
16. Please indicate below, by selecting the appropriate boxes, which healthcare practitioner refers patients with Transthyretin Amyloid Cardiomyopathy to your institution?
    - A general practitioner
    - A family physician
    - An internal medicine physician
    - A cardiologist
    - A neurologist
    - A hematologist
    - A geriatrician
    - A geneticists
17. **ATTITUDES AND BARRIERS TOWARD THE DIAGNOSIS OF THE DISEASE**
18. To what extent do you agree with each of the following statements?
    1. I am aware of the risk factors for Transthyretin Amyloid Cardiomyopathy (i.e., something that increases the chance of developing a disease)
    - Strongly Disagree
    - Disagree
    - Neutral
    - Agree
    - Strongly Agree
    1. I am aware of the symptoms of Transthyretin Amyloid Cardiomyopathy
    - Strongly Disagree
    - Disagree
    - Neutral
    - Agree
    - Strongly Agree
    1. Timely diagnosis of Transthyretin Amyloid Cardiomyopathy is uncommon
    - Strongly Disagree
    - Disagree
    - Neutral
    - Agree
    - Strongly Agree
    1. Lack of timely diagnosis of Transthyretin Amyloid Cardiomyopathy delays appropriate management
    - Strongly Disagree
    - Disagree
    - Neutral
    - Agree
    - Strongly Agree
19. Do you agree that each of the following findings would independently indicate referral to a specialized healthcare practitioner under the suspicion of Transthyretin Amyloid Cardiomyopathy?

| - - 1. Family history (mother/father) of heart or neurological disease | Yes | No |
| --- | --- | --- |
| - - 1. History of treatment-resistant hypertension | Yes | No |
| - - 1. History of non-responsivity to standard medications to heart failure management | Yes | No |
| - - 1. Coexistence of chronic kidney disease and heart failure (cardiorenal syndrome) | Yes | No |
| - - 1. A “natural cure” of hypertension, understood as the need for down-titration or discontinuation of anti-hypertensive therapy | Yes | No |
| - - 1. History of hospitalization due to pulmonary congestion | Yes | No |
| - - 1. Intolerance of beta blockade in newly diagnosed heart failure | Yes | No |
| - - 1. . History of heart failure with headaches | Yes | No |
| - - 1. . History of heart failure with bilateral carpal tunnel syndrome | Yes | No |
| - - 1. History of musculoskeletal pain | Yes | No |
| - - 1. History of heart failure with lumbar spinal stenosis | Yes | No |
| - - 1. History of common respiratory infections | Yes | No |
| - - 1. History of heart failure with spontaneous biceps tendon rupture | Yes | No |
| - - 1. History of acid reflux or gastroesophageal reflux disease | Yes | No |
| - - 1. History of heart failure with bilateral carpal tunnel syndrome, lumbar spinal stenosis, and spontaneous biceps tendon rupture | Yes | No |
| - - 1. History of anxiety or panic attacks | Yes | No |
| - - 1. Unexplained peripheral or autonomic neuropathy | Yes | No |
| - - 1. Unexplained weight loss | Yes | No |
| - - 1. Increased left ventricular wall thickness in the presence of a low-voltage ECG pattern | Yes | No |
| - - 1. Low-flow low-gradient aortic stenosis | Yes | No |
| - - 1. Unexplained increased left or right ventricular wall thickness | Yes | No |
| - - 1. Increased interventricular septal wall thickness (>12 mm) | Yes | No |
| - - 1. Persistent elevation in cardiac biomarkers (i.e., Troponin T and NTproBNP) | Yes | No |
| - - 1. Conduction system disease and arrhythmias, which occur years before the onset of heart failure | Yes | No |
| - - 1. Thickening of the heart muscle or increased echogenicity on echocardiogram without strain | Yes | No |
| - - 1. Restrictive filling pattern on echocardiogram without strain | Yes | No |
| - - 1. Increased left atrial size on echocardiogram without strain | Yes | No |
| - - 1. Pericardial effusion on echocardiogram without strain | Yes | No |
| - - 1. Abnormal ratios of apical to basal strain, or apical to basal plus mid-ventricular strain | Yes | No |

- 1. For the questions where you selected “Yes”, please rank the importance of recognizing this characteristic in patients with Transthyretin Amyloid Cardiomyopathy in order of relevance (0 for least relevance and 10 for highest relevance)._______________

1. Please answer according to the availability of the corresponding test:

|  | Is it available in your institution? | | Is it available through other means in your area (e.g., private practice)?  Ask if previous question = "No" | |
| --- | --- | --- | --- | --- |
| 1. Family history (mother/father) of heart or neurological disease | Yes | No | Yes | No |
| 1. History of treatment-resistant hypertension | Yes | No | Yes | No |
| 1. History of non-responsivity to standard medications to heart failure management | Yes | No | Yes | No |
| 1. Coexistence of chronic kidney disease and heart failure (cardiorenal syndrome) | Yes | No | Yes | No |
| 1. A “natural cure” of hypertension, understood as the need for down-titration or discontinuation of anti-hypertensive therapy | Yes | No | Yes | No |
| 1. History of hospitalization due to pulmonary congestion | Yes | No | Yes | No |
| 1. Intolerance of beta blockade in newly diagnosed heart failure | Yes | No | Yes | No |
| 1. . History of heart failure with headaches | Yes | No | Yes | No |
| 1. . History of heart failure with bilateral carpal tunnel syndrome | Yes | No | Yes | No |
| 1. History of musculoskeletal pain | Yes | No | Yes | No |
| 1. History of heart failure with lumbar spinal stenosis | Yes | No | Yes | No |
| 1. History of common respiratory infections | Yes | No | Yes | No |
| 1. History of heart failure with spontaneous biceps tendon rupture | Yes | No | Yes | No |
| 1. History of acid reflux or gastroesophageal reflux disease | Yes | No | Yes | No |

1. In the last 12 months, how many of the following advanced diagnostic procedures have been performed or ordered in your institution to confirm a diagnosis of Transthyretin Amyloid Cardiomyopathy (number of tests)?
   1. Bone Scintigraphy
   - None
   - <5
   - 5-15
   - 16-30
   - 31-45
   - >45
   1. Cardiac magnetic resonance imaging (CMRI)
   - None
   - <5
   - 5-15
   - 16-30
   - 31-45
   - >45
   1. Echocardiogram with strain
   - None
   - <5
   - 5-15
   - 16-30
   - 31-45
   - >45
   1. Echocardiogram without strain
   - None
   - <5
   - 5-15
   - 16-30
   - 31-45
   - >45
   1. Transthyretin Genetic testing
   - None
   - <5
   - 5-15
   - 16-30
   - 31-45
   - >45
   1. NT-proBNP
   - None
   - <5
   - 5-15
   - 16-30
   - 31-45
   - >45
   1. Serum/Urine Protein Electrophoresis
   - None
   - <5
   - 5-15
   - 16-30
   - 31-45
   - >45
   1. Free-Light Chain Assay (FLC)
   - None
   - <5
   - 5-15
   - 16-30
   - 31-45
   - >45
   1. Troponin T/I
   - None
   - <5
   - 5-15
   - 16-30
   - 31-45
   - >45
   1. Non-cardiac tissue biopsy
   - None
   - <5
   - 5-15
   - 16-30
   - 31-45
   - >45
   1. Cardiac biopsy
   - None
   - <5
   - 5-15
   - 16-30
   - 31-45
   - >45
   1. Immunohistochemistry for Transthyretin Amyloidosis
   - None
   - <5
   - 5-15
   - 16-30
   - 31-45
   - >45
   1. Mass spectrometry for Transthyretin Amyloidosis
   - None
   - <5
   - 5-15
   - 16-30
   - 31-45
   - >45
   1. Quantitative cardiac single photon emission computed tomography/positron emission tomography (SPECT/PET) using bone avid radiotracers
   - None
   - <5
   - 5-15
   - 16-30
   - 31-45
   - >45
2. When selecting a test to confirm the diagnosis of Transthyretin Amyloid Cardiomyopathy in order of priority, which one do you consider the highest priority?
   1. Bone Scintigraphy
   - Low priority
   - Medium priority
   - High priority
   1. Cardiac magnetic resonance imaging (CMRI)
   - Low priority
   - Medium priority
   - High priority
   1. Transthyretin Genetic testing
   - Low priority
   - Medium priority
   - High priority
   1. NT-proBNP
   - Low priority
   - Medium priority
   - High priority
   1. Serum/Urine Protein Electrophoresis
   - Low priority
   - Medium priority
   - High priority
   1. Free-Light Chain Assay (FLC)
   - Low priority
   - Medium priority
   - High priority
   1. Troponin T/I
   - Low priority
   - Medium priority
   - High priority
   1. Non-cardiac tissue biopsy
   - Low priority
   - Medium priority
   - High priority
   1. Cardiac biopsy
   - Low priority
   - Medium priority
   - High priority
   1. Mass spectrometry for Transthyretin Amyloidosis
   - Low priority
   - Medium priority
   - High priority
   1. Immunohistochemistry for Transthyretin Amyloidosis
   - Low priority
   - Medium priority
   - High priority
   1. Quantitative cardiac single photon emission computed tomography/positron emission tomography (SPECT/PET) using bone avid radiotracers
   - Low priority
   - Medium priority
   - High priority
   1. Is SPECT capture suggestive of intramyocardial deposit?
   - Yes
   - No
   1. Is SPECT done for diagnosing patients with Transthyretin Amyloid Cardiomyopathy in your institution?
   - Yes
   - No
3. Is there a clear algorithm / protocol for diagnosing patients with Transthyretin Amyloid Cardiomyopathy in your institution?
   - Yes
   - No
4. Is there a clear algorithm / protocol for diagnosing patients with Wild-type Transthyretin Amyloidosis in your institution?
   - Yes
   - No
5. Is there a clear algorithm / protocol for diagnosing patients with Hereditary Transthyretin Amyloidosis in your institution?
   - Yes
   - No
6. Is there a clear algorithm / protocol for diagnosing patients with Transthyretin Amyloid Polyneuropathy in your institution?
   - Yes
   - No
7. Is there a clear algorithm / protocol for diagnosing patients with Mixed phenotype Transthyretin Amyloidosis in your institution?
   - Yes
   - No
8. If there is a clear algorithm/protocol for diagnosing the aforementioned conditions in your institution, to what extent do you agree with each of the following statements?
   1. It is easy to follow
   - Strongly Disagree
   - Disagree
   - Neutral
   - Agree
   - Strongly Agree
   1. It includes a multidisciplinary approach
   - Strongly Disagree
   - Disagree
   - Neutral
   - Agree
   - Strongly Agree
   1. It is the same as for patients with Polyneuropathy
   - Strongly Disagree
   - Disagree
   - Neutral
   - Agree
   - Strongly Agree
9. If there is not a clear algorithm/protocol for diagnosing the aforementioned conditions in your institution, to what extent do you agree with each of the following statements?
   1. The diagnosis is based on the subjective experience of the corresponding physician
   - Strongly Disagree
   - Disagree
   - Neutral
   - Agree
   - Strongly Agree
   1. A clear algorithm/protocol is under development
   - Strongly Disagree
   - Disagree
   - Neutral
   - Agree
   - Strongly Agree
10. How much, if anything, would you say you know about the Clinical staging systems for Transthyretin Amyloid Cardiomyopathy
    - A great deal
    - A fair amount
    - Just a little
    - Heard of, know nothing about it
    - Never heard of it
11. How easily available is Transthyretin Amyloidosis genetic testing in your country?
    - It is not available
    - It is available through industry-sponsored support
    - It is available only in major centers
    - It is widely available
12. Regarding Transthyretin Amyloidosis genetic testing in your country, to what extent do you agree with each of the following statements?
    1. Although genetic testing is available, some physicians are unaware of its importance
    - Strongly Disagree
    - Disagree
    - Neutral
    - Agree
    - Strongly Agree
    1. Some centers still have barriers to outpatient care access
    - Strongly Disagree
    - Disagree
    - Neutral
    - Agree
    - Strongly Agree
    1. Some centers still have barriers to inpatient care access
    - Strongly Disagree
    - Disagree
    - Neutral
    - Agree
    - Strongly Agree
    1. Some centers still have barriers to genetic testing
    - Strongly Disagree
    - Disagree
    - Neutral
    - Agree
    - Strongly Agree
    1. Some centers still have barriers to genetic counseling
    - Strongly Disagree
    - Disagree
    - Neutral
    - Agree
    - Strongly Agree
13. Regarding the unmet needs in diagnosing patients with Transthyretin Amyloid Cardiomyopathy, to what extent do you agree with each of the following statements?
    1. There is a need to improve disease awareness among cardiologists
    - Strongly Disagree
    - Disagree
    - Neutral
    - Agree
    - Strongly Agree
    1. There is a need to improve disease awareness among physicians other than cardiologist
    - Strongly Disagree
    - Disagree
    - Neutral
    - Agree
    - Strongly Agree
    1. There are significant barriers regarding the access to sensitive diagnostic imaging tools
    - Strongly Disagree
    - Disagree
    - Neutral
    - Agree
    - Strongly Agree
    1. There are significant barriers regarding the access to specific diagnostic imaging tools
    - Strongly Disagree
    - Disagree
    - Neutral
    - Agree
    - Strongly Agree
    1. There are significant barriers regarding the access to sensitive diagnostic laboratory tools
    - Strongly Disagree
    - Disagree
    - Neutral
    - Agree
    - Strongly Agree
    1. There are significant barriers regarding the access to specific diagnostic laboratory tools
    - Strongly Disagree
    - Disagree
    - Neutral
    - Agree
    - Strongly Agree
    1. There are significant barriers regarding the access to risk-stratification tools
    - Strongly Disagree
    - Disagree
    - Neutral
    - Agree
    - Strongly Agree
    1. There are significant barriers regarding the access to biomarkers used to measure the progress of the disease
    - Strongly Disagree
    - Disagree
    - Neutral
    - Agree
    - Strongly Agree
    1. There is a need to improve knowledge and training when evaluating extra-cardiac manifestations
    - Strongly Disagree
    - Disagree
    - Neutral
    - Agree
    - Strongly Agree
    1. There is a need to improve knowledge and training when identifying Red Flags of the disease
    - Strongly Disagree
    - Disagree
    - Neutral
    - Agree
    - Strongly Agree
    1. There is a need to improve knowledge and training when identifying the different ages of disease onset
    - Strongly Disagree
    - Disagree
    - Neutral
    - Agree
    - Strongly Agree
    1. There is a need to improve knowledge when considering the genotype–phenotype interaction
    - Strongly Disagree
    - Disagree
    - Neutral
    - Agree
    - Strongly Agree
    1. There is a need to improve knowledge and training about carriers’ follow-up
    - Strongly Disagree
    - Disagree
    - Neutral
    - Agree
    - Strongly Agree
    1. There is a need to improve knowledge when considering the utility of image / biomarker in the follow-up of the disease
    - Strongly Disagree
    - Disagree
    - Neutral
    - Agree
    - Strongly Agree
14. **ATTITUDES AND BARRIERS TOWARD THE TREATMENT OF THE DISEASE (FORM 5)**
15. To what extent do you agree with each of the following statements?
    1. Transthyretin Amyloid Cardiomyopathy is an untreatable disease
    - Strongly Disagree
    - Disagree
    - Neutral
    - Agree
    - Strongly Agree
16. If local treatment protocols were conducted, what would they be based on?
    - International guidelines
    - National guidelines
    - Institutional preferences
17. In your opinion, how important is Real-World-Data when making treatment decisions?
    - Not important at all
    - Somewhat unimportant
    - Neutral
    - Somewhat important
    - Extremely important
18. Regarding the unmet needs in treating patients with Transthyretin Amyloid Cardiomyopathy, to what extent do you agree with each of the following statements
    1. Many patients face cost-related barriers to care
    - Strongly Disagree
    - Disagree
    - Neutral
    - Agree
    - Strongly Agree
    1. There are significant barriers regarding the access to Tafamidis
    - Strongly Disagree
    - Disagree
    - Neutral
    - Agree
    - Strongly Agree
    1. There is a need to tailor the treatment to the individual
    - Strongly Disagree
    - Disagree
    - Neutral
    - Agree
    - Strongly Agree
    1. There is a lack of guidance for evaluating progression criteria
    - Strongly Disagree
    - Disagree
    - Neutral
    - Agree
    - Strongly Agree
    1. There is a lack of evidence regarding the benefit of current therapies for a severe and progressive disease with cardiac/renal involvement
    - Strongly Disagree
    - Disagree
    - Neutral
    - Agree
    - Strongly Agree
    1. The treatment decision is a shared decisionmaking process between the physician and/or multidisciplinary team and patient
    - Strongly Disagree
    - Disagree
    - Neutral
    - Agree
    - Strongly Agree
19. What factor(s) influence whether or not you prescribe a specific treatment for a given patient?
    - Progression of disease
    - Patient preference
    - Patients ability to pay for the test
    - Insurer ability to pay for the test
    - Availability of particular treatment needed where I practice
    - Local Guidelines
    - International Guidelines
    - Time to obtain test results
    - Time to obtain test results
20. What would help you to use a specific treatment more often in your practice?
    - Information and tools to help patients understand treatment effect
    - Training for myself
    - Greater availability of treatments where I practice
    - Improved reimbursement conditions for treatments
    - Overall reduction in cost of treatments
    - Reduction in time taken to receive results
    - Inclusion in local/hospital guidelines
    - Nothing would help me use a specific treatment more often in my practice
21. **ATTITUDES AND BARRIERS TOWARD THE FOLLOW-UP OF THE DISEASE (FORM 6)**
22. Which of the following, if any, do you typically perform as part of the follow-up of Transthyretin Amyloid Cardiomyopathy patients?
    - NYHA class evaluation
    - Cardiomyopathy Questionnaire (Kansas City) (KCCQ-12)
    - EQ-5D tool
    - 6-min walk test
    - NT-proBNP
    - Troponin (high-sensitivity) assay
    - Echocardiography - LV measures wall thickness/mass
    - Echocardiography - Systolic function measurements
    - Echocardiography - Diastolic function measurements
    - ECG/Holter ECG
23. Do you think your patients feel fully informed and understand their diseases, treatment, and prognosis when it is explained by you?
    - Always
    - Sometimes
    - Rarely
    - Never
24. What sources of information, if any, are available to your patients about their disease?
    - Family, friends
    - Nurses
    - Support groups
    - Internet sites o Newspaper or magazine articles
    - Hospital patient information leaflet
25. Would you be willing for Pfizer to re-contact you in the next 12 months about this survey?
    - Yes
    - No
