# Supplementary material for: Awareness, attitudes, and barriers toward Transthyretin Amyloid Cardiomyopathy in Latin America: A questionnaire-based cross-sectional study
Source: PLoS One. 2026 Jun 26;21(6):e0351224. doi: 10.1371/journal.pone.0351224 (PMC13309013; doi:10.1371/journal.pone.0351224)
Supplement: Attachment — Submitted filename: Response to Reviewers.docx [file pone.0351224.s003.docx]

**General Response**

We thank the Editor and both reviewers for their careful evaluation of our manuscript and for their constructive and detailed feedback. All comments have been addressed point by point, and changes have been incorporated into the revised manuscript. Below we provide a detailed response to each comment.

**Reviewer #1**

We thank Reviewer #1 for the positive assessment of the relevance of our study and for the insightful comments that helped strengthen the manuscript.

**Minor Revisions**

**Comment:**
“Amyloidosis Cardiomyopathy (ATTRCA) is incorrect. The correct term is Transthyretin Amyloid Cardiomyopathy (ATTRCM).”

**Response:**
We agree with the reviewer.

**Change made:**
All instances of “ATTRCA” were replaced with **“ATTRCM”** throughout the manuscript.

**Comment:**
“In line 105, in addition to reference 7, a recent review describing all identified mutations in the TTR gene should be included.”

**Response:**
Thank you for this valuable suggestion.

**Change made:**
The suggested review article (DOI: 10.1080/10408363.2024.2350379) was added to the Introduction.

**Comment:**
“According to Amyloid Nomenclature 2024, abbreviations for wild-type and hereditary ATTR should be TTRwt and ATTRv.”

**Response:**
We appreciate this clarification.

**Change made:**
All terminology related to amyloid subtypes was revised to align with the 2024 Amyloid Nomenclature, consistently using **ATTRwt** and **ATTRv**.

**Comment:**
“‘CARI’ is used without definition.”

**Response:**
We agree.

**Change made:**
The abbreviation was removed, as it was used only once.

**Major Revisions**

**Comment:**
“The authors should consider providing the full questionnaire as supplementary material.”

**Response:**
We agree that this improves transparency.

**Change made:**
The full questionnaire has been included as **Supplementary Material**.

**Comment:**
“The manuscript would benefit from adherence to the STROBE guidelines for cross-sectional studies.”

**Response:**
We fully agree.

**Change made:**
The Methods section was revised to explicitly state adherence to the **STROBE guidelines**, and relevant adjustments were made throughout the manuscript.

**Comment:**
“The statistical analysis requires clearer justification and description.”

**Response:**
We appreciate this important observation.

**Change made:**
The Statistical Analysis section now clarifies that the study is **primarily descriptive**, with **exploratory, hypothesis-generating inferential analyses**. We explicitly describe the rationale for the analyses, variables included, and the absence of multiple comparison adjustments, justified by the exploratory nature of the study. These clarifications are also reflected in the Limitations section.

**Comment:**
“Clarify the ethics approval in light of the Declaration of Helsinki.”

**Response:**
We thank the reviewer for highlighting this important point.

**Change made:**
The Ethics section was revised to clarify that the study involved an anonymous, non-interventional survey of healthcare professionals, without patient data, and therefore did not require formal ethics committee approval, while still adhering to the ethical principles of the Declaration of Helsinki.

**Comment:**
“A more in-depth discussion on translation into clinical or policy interventions would increase relevance.”

**Response:**
We agree.

**Change made:**
The Discussion was expanded to better link identified gaps in awareness, diagnosis, and access to actionable clinical and policy implications.

**Comment:**
“Figure 1 appears to be of low resolution.”

**Response:**
Thank you for noting this.

**Change made:**
A higher-resolution version of Figure 1 was uploaded.

**Comment:**
“Some conclusions overstate awareness levels; English revision is recommended.”

**Response:**
We fully agree.

**Change made:**
The Conclusions were revised to align strictly with the descriptive and self-reported nature of the data, and comprehensive English language editing was performed throughout the manuscript.

**Reviewer #2**

We thank Reviewer #2 for the thoughtful evaluation and helpful comments.

**Comment:**
“More details are needed on how physicians were informed about the survey.”

**Response:**
We agree.

**Change made:**
The Methods section was expanded to describe physician recruitment, dissemination channels, voluntary participation, handling of incomplete surveys, and potential selection bias, which is now acknowledged in the Limitations.

**Comment:**
“There are some typos, but the manuscript is easy to read.”

**Response:**
We appreciate this feedback.

**Change made:**
All typographical and grammatical errors identified were corrected as part of the comprehensive language revision.
