# Supplementary material for: Awareness, attitudes, and barriers toward Transthyretin Amyloid Cardiomyopathy in Latin America: A questionnaire-based cross-sectional study
Source: PLoS One. 2026 Jun 26;21(6):e0351224. doi: 10.1371/journal.pone.0351224 (PMC13309013; doi:10.1371/journal.pone.0351224)
Supplement: Peer review history

# Peer review: Decision Letter 0

**DOI:** 10.1371/journal.pone.0351224.r001

**Authors:** Roberto Magalhães Saraiva

12 Apr 2026

PONE-D-26-09243

Awareness, attitudes, and barriers toward Transthyretin Amyloid Cardiomyopathy in Latin America: A questionnaire-based cross-sectional study

PLOS One

Dear Dr. Silva Julian,

Thank you for submitting your manuscript to PLOS ONE. After careful consideration, we feel that it has merit but does not fully meet PLOS ONE’s publication criteria as it currently stands. Therefore, we invite you to submit a revised version of the manuscript that addresses all points raised during the review process.

Please submit your revised manuscript by May 27 2026 11:59PM. If you will need more time than this to complete your revisions, please reply to this message or contact the journal office at plosone@plos.org. When you're ready to submit your revision, log on to https://www.editorialmanager.com/pone/ and select the 'Submissions Needing Revision' folder to locate your manuscript file.

Please include the following items when submitting your revised manuscript:

- A letter that responds to each point raised by the academic editor and reviewer(s). You should upload this letter as a separate file labeled 'Response to Reviewers'.
- A marked-up copy of your manuscript that highlights changes made to the original version. You should upload this as a separate file labeled 'Revised Manuscript with Track Changes'.
- An unmarked version of your revised paper without tracked changes. You should upload this as a separate file labeled 'Manuscript'.

If you would like to make changes to your financial disclosure, please include your updated statement in your cover letter. Guidelines for resubmitting your figure files are available below the reviewer comments at the end of this letter.

If applicable, we recommend that you deposit your laboratory protocols in protocols.io to enhance the reproducibility of your results. Protocols.io assigns your protocol its own identifier (DOI) so that it can be cited independently in the future. For instructions see: https://journals.plos.org/plosone/s/submission-guidelines#loc-laboratory-protocols. Additionally, PLOS ONE offers an option for publishing peer-reviewed Lab Protocol articles, which describe protocols hosted on protocols.io. Read more information on sharing protocols at https://plos.org/protocols?utm_medium=editorial-email&utm_source=authorletters&utm_campaign=protocols.

As the corresponding author, your ORCID iD is verified in the submission system and will appear in the published article. PLOS supports the use of ORCID, and we encourage all coauthors to register for an ORCID iD and use it as well. Please encourage your coauthors to verify their ORCID iD within the submission system before final acceptance, as unverified ORCID iDs will not appear in the published article. *Only*  the individual author can complete the verification step; PLOS staff *cannot*  verify ORCID iDs on behalf of authors.

We look forward to receiving your revised manuscript.

Kind regards,

Roberto Magalhães Saraiva, MD, PhD

Academic Editor

PLOS One

Journal Requirements:

When submitting your revision, we need you to address these additional requirements.

1. Please ensure that your manuscript meets PLOS ONE's style requirements, including those for file naming. The PLOS ONE style templates can be found at

https://journals.plos.org/plosone/s/file?id=wjVg/PLOSOne_formatting_sample_main_body.pdf and https://journals.plos.org/plosone/s/file?id=ba62/PLOSOne_formatting_sample_title_authors_affiliations.pdf

2. Please provide additional details regarding participant consent. In the ethics statement in the Methods and online submission information, please ensure that you have specified (1) whether consent was informed and (2) what type you obtained (for instance, written or verbal, and if verbal, how it was documented and witnessed). If your study included minors, state whether you obtained consent from parents or guardians. If the need for consent was waived by the ethics committee, please include this information.

If you are reporting a retrospective study of medical records or archived samples, please ensure that you have discussed whether all data were fully anonymized before you accessed them and/or whether the IRB or ethics committee waived the requirement for informed consent. If patients provided informed written consent to have data from their medical records used in research, please include this information.

3. Thank you for stating the following financial disclosure:

[This study was fund by Pfizer. The sponsors were not directly involved in the study, meaning that they had no role in the study design, data collection, and report writing.].

Please state what role the funders took in the study.  If the funders had no role, please state: "The funders had no role in study design, data collection and analysis, decision to publish, or preparation of the manuscript."

If this statement is not correct you must amend it as needed.

Please include this amended Role of Funder statement in your cover letter; we will change the online submission form on your behalf.

4. Thank you for stating the following in the Financial Disclosure section:

[This study was fund by Pfizer. The sponsors were not directly involved in the study, meaning that they had no role in the study design, data collection, and report writing.].

We note that you received funding from a commercial source: Pfizer Inc.

Please provide an amended Competing Interests Statement that explicitly states this commercial funder, along with any other relevant declarations relating to employment, consultancy, patents, products in development, marketed products, etc.

Within this Competing Interests Statement, please confirm that this does not alter your adherence to all PLOS ONE policies on sharing data and materials by including the following statement: "This does not alter our adherence to PLOS ONE policies on sharing data and materials.” (as detailed online in our guide for authors http://journals.plos.org/plosone/s/competing-interests).  If there are restrictions on sharing of data and/or materials, please state these. Please note that we cannot proceed with consideration of your article until this information has been declared.

Please include your amended Competing Interests Statement within your cover letter. We will change the online submission form on your behalf.

5. Thank you for stating the following in the Competing Interests section:

[The authors have declared that no competing interests exist.].

We note that one or more of the authors are employed by commercial companies: Pfizer Inc., IQVIA Inc., Clinica Bupa, Clinica Iberoamericana.

1. Please provide an amended Funding Statement declaring this commercial affiliation, as well as a statement regarding the Role of Funders in your study. If the funding organization did not play a role in the study design, data collection and analysis, decision to publish, or preparation of the manuscript and only provided financial support in the form of authors' salaries and/or research materials, please review your statements relating to the author contributions, and ensure you have specifically and accurately indicated the role(s) that these authors had in your study. You can update author roles in the Author Contributions section of the online submission form.

Please also include the following statement within your amended Funding Statement.

“The funder provided support in the form of salaries for authors [insert relevant initials], but did not have any additional role in the study design, data collection and analysis, decision to publish, or preparation of the manuscript. The specific roles of these authors are articulated in the ‘author contributions’ section.”

If your commercial affiliation did play a role in your study, please state and explain this role within your updated Funding Statement.

2. Please also provide an updated Competing Interests Statement declaring this commercial affiliation along with any other relevant declarations relating to employment, consultancy, patents, products in development, or marketed products, etc.

Within your Competing Interests Statement, please confirm that this commercial affiliation does not alter your adherence to all PLOS ONE policies on sharing data and materials by including the following statement: "This does not alter our adherence to PLOS ONE policies on sharing data and materials.” (as detailed online in our guide for authors http://journals.plos.org/plosone/s/competing-interests) . If this adherence statement is not accurate and there are restrictions on sharing of data and/or materials, please state these. Please note that we cannot proceed with consideration of your article until this information has been declared.

Please include both an updated Funding Statement and Competing Interests Statement in your cover letter. We will change the online submission form on your behalf.

6. We note that you have indicated that there are restrictions to data sharing for this study. For studies involving human research participant data or other sensitive data, we encourage authors to share de-identified or anonymized data. However, when data cannot be publicly shared for ethical reasons, we allow authors to make their data sets available upon request. For information on unacceptable data access restrictions, please see http://journals.plos.org/plosone/s/data-availability#loc-unacceptable-data-access-restrictions.

Before we proceed with your manuscript, please address the following prompts:

a) If there are ethical or legal restrictions on sharing a de-identified data set, please explain them in detail (e.g., data contain potentially identifying or sensitive patient information, data are owned by a third-party organization, etc.) and who has imposed them (e.g., a Research Ethics Committee or Institutional Review Board, etc.). Please also provide contact information for a data access committee, ethics committee, or other institutional body to which data requests may be sent.

b) If there are no restrictions, please upload the minimal anonymized data set necessary to replicate your study findings to a stable, public repository and provide us with the relevant URLs, DOIs, or accession numbers. Please see http://www.bmj.com/content/340/bmj.c181.long for guidelines on how to de-identify and prepare clinical data for publication. For a list of recommended repositories, please see https://journals.plos.org/plosone/s/recommended-repositories. You also have the option of uploading the data as Supporting Information files, but we would recommend depositing data directly to a data repository if possible.

Please update your Data Availability statement in the submission form accordingly.

7. We note that your Data Availability Statement is currently as follows: [All relevant data are within the manuscript and its Supporting Information files.]

Please confirm at this time whether or not your submission contains all raw data required to replicate the results of your study. Authors must share the “minimal data set” for their submission. PLOS defines the minimal data set to consist of the data required to replicate all study findings reported in the article, as well as related metadata and methods (https://journals.plos.org/plosone/s/data-availability#loc-minimal-data-set-definition).

For example, authors should submit the following data:

- The values behind the means, standard deviations and other measures reported;

- The values used to build graphs;

- The points extracted from images for analysis.

Authors do not need to submit their entire data set if only a portion of the data was used in the reported study.

If your submission does not contain these data, please either upload them as Supporting Information files or deposit them to a stable, public repository and provide us with the relevant URLs, DOIs, or accession numbers. For a list of recommended repositories, please see https://journals.plos.org/plosone/s/recommended-repositories.

If there are ethical or legal restrictions on sharing a de-identified data set, please explain them in detail (e.g., data contain potentially sensitive information, data are owned by a third-party organization, etc.) and who has imposed them (e.g., an ethics committee). Please also provide contact information for a data access committee, ethics committee, or other institutional body to which data requests may be sent. If data are owned by a third party, please indicate how others may request data access.

8. Please upload a new copy of Figure 1 as the detail is not clear. Please follow the link for more information:  https://journals.plos.org/plosone/s/figures

If the reviewer comments include a recommendation to cite specific previously published works, please review and evaluate these publications to determine whether they are relevant and should be cited. There is no requirement to cite these works unless the editor has indicated otherwise.

[Note: HTML markup is below. Please do not edit.]

Reviewers' comments:

Reviewer's Responses to Questions

**Comments to the Author**

1. Is the manuscript technically sound, and do the data support the conclusions?

The manuscript must describe a technically sound piece of scientific research with data that supports the conclusions. Experiments must have been conducted rigorously, with appropriate controls, replication, and sample sizes. The conclusions must be drawn appropriately based on the data presented.

Reviewer #1: Yes

Reviewer #2: Yes

**********

2. Has the statistical analysis been performed appropriately and rigorously?

Reviewer #1: No

Reviewer #2: Yes

**********

3. Have the authors made all data underlying the findings in their manuscript fully available?

The PLOS Data policy requires authors to make all data underlying the findings described in their manuscript fully available without restriction, with rare exception (please refer to the Data Availability Statement in the manuscript PDF file). The data should be provided as part of the manuscript or its supporting information, or deposited to a public repository. For example, in addition to summary statistics, the data points behind means, medians and variance measures should be available. If there are restrictions on publicly sharing data—e.g. participant privacy or use of data from a third party—those must be specified.

Reviewer #1: No

Reviewer #2: No

**********

4. Is the manuscript presented in an intelligible fashion and written in standard English?

PLOS ONE does not copyedit accepted manuscripts, so the language in submitted articles must be clear, correct, and unambiguous. Any typographical or grammatical errors should be corrected at revision, so please note any specific errors here.

Reviewer #1: No

Reviewer #2: Yes

**********

5. Review Comments to the Author

Please use the space provided to explain your answers to the questions above. You may also include additional comments for the author, including concerns about dual publication, research ethics, or publication ethics. (Please upload your review as an attachment if it exceeds 20,000 characters)

Reviewer #1: This manuscript presents a cross-sectional, questionnaire-based study assessing awareness, attitudes, and perceived barriers related to transthyretin amyloid cardiomyopathy (ATTR-CM) among physicians across multiple Latin American countries. The study addresses an important and underexplored topic, given the challenges associated with the recognition, diagnosis, and management of ATTR-CM in real-world clinical settings, particularly in regions with heterogeneous healthcare systems. By collecting data from a relatively large and diverse sample of healthcare professionals, the authors provide insights into current clinical practices, identify gaps in knowledge and access to diagnostic tools and treatments, and highlight potential areas for improvement in patient care. Overall, the study contributes valuable regional data that may help inform future educational strategies and healthcare planning initiatives.

However, the manuscript has significant methodological, reporting, and clarity issues that currently limit its scientific robustness and interpretability.

Minor revisions:

- “Amyloidosis Cardiomyopathy (ATTR-CA)” is incorrect. The correct term is “Transthyretin Amyloid Cardiomyopathy,” with the abbreviation ATTR-CM. Please revise this throughout the manuscript.

- In line 105, in addition to reference 7, the recent review article describing all identified mutations in the TTR gene should be included (DOI: 10.1080/10408363.2024.2350379).

- In line 123, according to the Amyloid Nomenclature 2024 (DOI: 10.1080/13506129.2024.2405948), the abbreviation for wild-type transthyretin amyloidosis should be TTRwt, and for hereditary ATTR amyloidosis, ATTRv. Please revise accordingly.

- In line 206, “CARI” is used without definition and should be defined at first mention.

Major revisions:

1) The authors should consider providing the full questionnaire as supplementary material to enhance transparency and facilitate the assessment of the study instrument.

2) The manuscript would benefit from adherence to the STROBE guidelines for cross-sectional studies, which would strengthen the transparency, completeness, and overall quality of reporting.

3) The statistical analysis also requires clearer justification and description. Although the study is presented as primarily descriptive, the inclusion of inferential analyses (e.g., ANOVA and multivariate approaches) suggests a more analytical framework. Clarifying the rationale for these analyses, the variables included in the models, and whether any adjustments for multiple comparisons were performed would strengthen the methodological rigor.

4) The authors state that formal approval from an institutional review board or independent ethics committee was not required due to the nature of the study. However, the authors also indicate that the study was conducted in accordance with the Declaration of Helsinki, which explicitly requires prior review by an independent ethics committee for studies involving human participants. Therefore, it would be helpful to clarify how these statements should be reconciled.

5) A more in-depth analysis of how the findings can be translated into concrete clinical or policy interventions would increase the practical relevance of the study. For example, detailing how the identified knowledge and access gaps could guide targeted educational initiatives, diagnostic flows, or improvements to each country's healthcare system would add value to the discussion.

6) Figure 1 appears to be of low resolution, which makes several elements difficult to read and limits the interpretation of the presented data. The authors are encouraged to provide a higher-resolution version of this figure.

7) Some conclusions appear to overstate the level of awareness and should be more closely aligned with the descriptive nature of the data. For example, statements suggesting “moderate to high awareness” in certain countries (e.g., Brazil and Mexico) are not fully supported by the reported findings, where a substantial proportion of physicians still report limited knowledge of ATTR-CM. Similarly, while the study identifies variability in diagnostic practices and access to tools, the conclusions sometimes imply a higher level of clinical preparedness than the results suggest, particularly given that a large proportion of respondents reported managing few or no patients with ATTR-CM in the past year. Therefore, the authors are encouraged to ensure that the conclusions more strictly reflect the descriptive and self-reported nature of the survey data.

8) The manuscript would benefit from careful revision of the English language. While the overall content is understandable, there are some grammatical mistakes. A thorough language editing is widely recommended.

Reviewer #2: This paper reports the results of an online cross sectional survey of internal medicine, cardiology and neurology physicians managing cardiac amyloidosis conducted in 8 latin american countries. The results indicate substantial knowledge gaps, lack of resourcces, delay in diagnosis , and heterogeneity of management of the condition.

The data are original but of limited novelty since the concept has been shown in other countries including the US. These data are probably of limited interest to the broader readership beyond Latin America.

The methodology is valid, but more details are needed on how physicians were made aware of survey and who responded, to understand the inevitable biases inherent in such studies. The conclusion are forthcoming from the data they present. There are some typos, but overall the manuscript is presented well and easy to read.

**********

6. PLOS authors have the option to publish the peer review history of their article (what does this mean?). If published, this will include your full peer review and any attached files.

If you choose “no”, your identity will remain anonymous but your review may still be made public.

**Do you want your identity to be public for this peer review?**  For information about this choice, including consent withdrawal, please see our Privacy Policy.

Reviewer #1: No

Reviewer #2: No

**********

[NOTE: If reviewer comments were submitted as an attachment file, they will be attached to this email and accessible via the submission site. Please log into your account, locate the manuscript record, and check for the action link "View Attachments". If this link does not appear, there are no attachment files.]

To ensure your figures meet our technical requirements, please review our figure guidelines: https://journals.plos.org/plosone/s/figures

You may also use PLOS’s free figure tool, NAAS, to help you prepare publication quality figures: https://journals.plos.org/plosone/s/figures#loc-tools-for-figure-preparation.

NAAS will assess whether your figures meet our technical requirements by comparing each figure against our figure specifications.

# Author comment: Author response to Decision Letter 1

**DOI:** 10.1371/journal.pone.0351224.r002

1 May 2026

General Response

We thank the Editor and both reviewers for their careful evaluation of our manuscript and for their constructive and detailed feedback. All comments have been addressed point by point, and changes have been incorporated into the revised manuscript. Below we provide a detailed response to each comment.

________________________________________

Reviewer #1

We thank Reviewer #1 for the positive assessment of the relevance of our study and for the insightful comments that helped strengthen the manuscript.

Minor Revisions

Comment:

“Amyloidosis Cardiomyopathy (ATTRCA) is incorrect. The correct term is Transthyretin Amyloid Cardiomyopathy (ATTRCM).”

Response:

We agree with the reviewer.

Change made:

All instances of “ATTRCA” were replaced with “ATTRCM” throughout the manuscript.

________________________________________

Comment:

“In line 105, in addition to reference 7, a recent review describing all identified mutations in the TTR gene should be included.”

Response:

Thank you for this valuable suggestion.

Change made:

The suggested review article (DOI: 10.1080/10408363.2024.2350379) was added to the Introduction.

________________________________________

Comment:

“According to Amyloid Nomenclature 2024, abbreviations for wild-type and hereditary ATTR should be TTRwt and ATTRv.”

Response:

We appreciate this clarification.

Change made:

All terminology related to amyloid subtypes was revised to align with the 2024 Amyloid Nomenclature, consistently using ATTRwt and ATTRv.

________________________________________

Comment:

“‘CARI’ is used without definition.”

Response:

We agree.

Change made:

The abbreviation was removed, as it was used only once.

________________________________________

Major Revisions

Comment:

“The authors should consider providing the full questionnaire as supplementary material.”

Response:

We agree that this improves transparency.

Change made:

The full questionnaire has been included as Supplementary Material.

________________________________________

Comment:

“The manuscript would benefit from adherence to the STROBE guidelines for cross-sectional studies.”

Response:

We fully agree.

Change made:

The Methods section was revised to explicitly state adherence to the STROBE guidelines, and relevant adjustments were made throughout the manuscript.

________________________________________

Comment:

“The statistical analysis requires clearer justification and description.”

Response:

We appreciate this important observation.

Change made:

The Statistical Analysis section now clarifies that the study is primarily descriptive, with exploratory, hypothesis-generating inferential analyses. We explicitly describe the rationale for the analyses, variables included, and the absence of multiple comparison adjustments, justified by the exploratory nature of the study. These clarifications are also reflected in the Limitations section.

________________________________________

Comment:

“Clarify the ethics approval in light of the Declaration of Helsinki.”

Response:

We thank the reviewer for highlighting this important point.

Change made:

The Ethics section was revised to clarify that the study involved an anonymous, non-interventional survey of healthcare professionals, without patient data, and therefore did not require formal ethics committee approval, while still adhering to the ethical principles of the Declaration of Helsinki.

________________________________________

Comment:

“A more in-depth discussion on translation into clinical or policy interventions would increase relevance.”

Response:

We agree.

Change made:

The Discussion was expanded to better link identified gaps in awareness, diagnosis, and access to actionable clinical and policy implications.

________________________________________

Comment:

“Figure 1 appears to be of low resolution.”

Response:

Thank you for noting this.

Change made:

A higher-resolution version of Figure 1 was uploaded.

________________________________________

Comment:

“Some conclusions overstate awareness levels; English revision is recommended.”

Response:

We fully agree.

Change made:

The Conclusions were revised to align strictly with the descriptive and self-reported nature of the data, and comprehensive English language editing was performed throughout the manuscript.

________________________________________

Reviewer #2

We thank Reviewer #2 for the thoughtful evaluation and helpful comments.

Comment:

“More details are needed on how physicians were informed about the survey.”

Response:

We agree.

Change made:

The Methods section was expanded to describe physician recruitment, dissemination channels, voluntary participation, handling of incomplete surveys, and potential selection bias, which is now acknowledged in the Limitations.

________________________________________

Comment:

“There are some typos, but the manuscript is easy to read.”

Response:

We appreciate this feedback.

Change made:

All typographical and grammatical errors identified were corrected as part of the comprehensive language revision.

Attachment Submitted filename: Response to Reviewers.docx

# Peer review: Decision Letter 1

**DOI:** 10.1371/journal.pone.0351224.r003

**Authors:** Roberto Magalhães Saraiva

25 May 2026

Awareness, attitudes, and barriers toward Transthyretin Amyloid Cardiomyopathy in Latin America: A questionnaire-based cross-sectional study

PONE-D-26-09243R1

Dear Dr. Silva Julian,

We’re pleased to inform you that your manuscript has been judged scientifically suitable for publication and will be formally accepted for publication once it meets all outstanding technical requirements.

Within one week, you’ll receive an e-mail detailing the required amendments. When these have been addressed, you’ll receive a formal acceptance letter and your manuscript will be scheduled for publication.

An invoice will be generated when your article is formally accepted. Please note, if your institution has a publishing partnership with PLOS and your article meets the relevant criteria, all or part of your publication costs will be covered. Please make sure your user information is up-to-date by logging into Editorial Manager at Editorial Manager® and clicking the ‘Update My Information' link at the top of the page. For questions related to billing, please contact billing support.

If your institution or institutions have a press office, please notify them about your upcoming paper to help maximize its impact. If they’ll be preparing press materials, please inform our press team as soon as possible -- no later than 48 hours after receiving the formal acceptance. Your manuscript will remain under strict press embargo until 2 pm Eastern Time on the date of publication. For more information, please contact onepress@plos.org.

Kind regards,

Roberto Magalhães Saraiva, MD, PhD

Academic Editor

PLOS One

Additional Editor Comments (optional):

Reviewers' comments:

Reviewer's Responses to Questions

**Comments to the Author**

1. If the authors have adequately addressed your comments raised in a previous round of review and you feel that this manuscript is now acceptable for publication, you may indicate that here to bypass the “Comments to the Author” section, enter your conflict of interest statement in the “Confidential to Editor” section, and submit your "Accept" recommendation.

Reviewer #1: All comments have been addressed

Reviewer #2: All comments have been addressed

**********

2. Is the manuscript technically sound, and do the data support the conclusions?

The manuscript must describe a technically sound piece of scientific research with data that supports the conclusions. Experiments must have been conducted rigorously, with appropriate controls, replication, and sample sizes. The conclusions must be drawn appropriately based on the data presented.

Reviewer #1: Yes

Reviewer #2: Yes

**********

3. Has the statistical analysis been performed appropriately and rigorously?

Reviewer #1: Yes

Reviewer #2: Yes

**********

4. Have the authors made all data underlying the findings in their manuscript fully available?

The PLOS Data policy requires authors to make all data underlying the findings described in their manuscript fully available without restriction, with rare exception (please refer to the Data Availability Statement in the manuscript PDF file). The data should be provided as part of the manuscript or its supporting information, or deposited to a public repository. For example, in addition to summary statistics, the data points behind means, medians and variance measures should be available. If there are restrictions on publicly sharing data—e.g. participant privacy or use of data from a third party—those must be specified.

Reviewer #1: Yes

Reviewer #2: Yes

**********

5. Is the manuscript presented in an intelligible fashion and written in standard English?

PLOS ONE does not copyedit accepted manuscripts, so the language in submitted articles must be clear, correct, and unambiguous. Any typographical or grammatical errors should be corrected at revision, so please note any specific errors here.

Reviewer #1: Yes

Reviewer #2: Yes

**********

6. Review Comments to the Author

Please use the space provided to explain your answers to the questions above. You may also include additional comments for the author, including concerns about dual publication, research ethics, or publication ethics. (Please upload your review as an attachment if it exceeds 20,000 characters)

Reviewer #1: (No Response)

Reviewer #2: (No Response)

**********

7. PLOS authors have the option to publish the peer review history of their article (what does this mean?). If published, this will include your full peer review and any attached files.

If you choose “no”, your identity will remain anonymous but your review may still be made public.

**Do you want your identity to be public for this peer review?**  For information about this choice, including consent withdrawal, please see our Privacy Policy.

Reviewer #1: No

Reviewer #2: No

**********

# Editor report: Acceptance letter

**DOI:** 10.1371/journal.pone.0351224.r004

**Authors:** Roberto Magalhães Saraiva

PONE-D-26-09243R1

PLOS One

Dear Dr. Silva Julian,

I'm pleased to inform you that your manuscript has been deemed suitable for publication in PLOS One. Congratulations! Your manuscript is now being handed over to our production team.

At this stage, our production department will prepare your paper for publication. This includes ensuring the following:

* All references, tables, and figures are properly cited

* All relevant supporting information is included in the manuscript submission,

* There are no issues that prevent the paper from being properly typeset

You will receive further instructions from the production team, including instructions on how to review your proof when it is ready. Please keep in mind that we are working through a large volume of accepted articles, so please give us a few days to review your paper and let you know the next and final steps.

Lastly, if your institution or institutions have a press office, please let them know about your upcoming paper now to help maximize its impact. If they'll be preparing press materials, please inform our press team within the next 48 hours. Your manuscript will remain under strict press embargo until 2 pm Eastern Time on the date of publication. For more information, please contact onepress@plos.org.

You will receive an invoice from PLOS for your publication fee after your manuscript has reached the completed accept phase. If you receive an email requesting payment before acceptance or for any other service, this may be a phishing scheme. Learn how to identify phishing emails and protect your accounts at https://explore.plos.org/phishing.

If we can help with anything else, please email us at customercare@plos.org.

Thank you for submitting your work to PLOS ONE and supporting open access.

Kind regards,

PLOS ONE Editorial Office Staff

on behalf of

Dr. Roberto Magalhães Saraiva

Academic Editor

PLOS One
